# Viral reservoir characteristics in lymphoid tissues of HIV-1 elite controllers

**DOI:** 10.1172/jci.insight.197308

**Published:** 2025-10-28

**Authors:** Samantha K. Marzi, Chloé M. Naasz, Leah Carrere, Carmen Gasca Capote, Isabelle C. Roseto, Ce Gao, Matthias Cavassini, Andrea Mastrangelo, Mathias Lichterfeld, Matthieu Perreau, Xu G. Yu

**Affiliations:** 1 Ragon Institute of MGB, MIT and Harvard and Infectious Disease Division, Brigham and Women’s Hospital, Boston, Massachusetts, USA.; 2 Instituto de Biomedicina de Sevilla and Spanish National Research Council, Seville, Spain.; 3 Division of Infectious Diseases and; 4 Division of Immunology and Allergy, Universite de Lausanne and Centre Hospitalier Universitaire Vaudois, Lausanne, Switzerland.

**Keywords:** AIDS/HIV, Infectious disease, Virology, Molecular biology

## Abstract

Elite controllers (ECs) maintain undetectable levels of plasma viremia in the absence of treatment, but small reservoirs of replication-competent proviruses persist in the vast majority of these persons. We longitudinally studied paired blood and inguinal lymph node samples (LNMC) from 2 ECs to better characterize distinguishing features of viral reservoir cell dynamics in ECs. In both participants, we observed a 7- to 10-fold lower frequency of intact proviruses in LNMC samples relative to reservoir cells circulating in blood. The landscape of intact proviruses in both tissue compartments was dominated by shared large clones that were frequently integrated in noncoding DNA regions, but the frequency and diversity of intact proviruses was more limited in LNMCs. Of note, over 9–10 years of longitudinal follow-up, a 3- to 18-fold reduction of intact proviruses was observed. Together, these data support the hypothesis that viral reservoirs in EC blood and lymphoid tissues are under strong, likely immune-mediated selection pressure.

## Abstract 2

Marzi and Naasz et al demonstrate that genome-intact proviruses from elite controllers are less frequent in lymphoid tissues compared to blood and longitudinally decline.

## Introduction

In the absence of antiretroviral treatment, HIV-1 infection typically leads to progressive depletion of CD4^+^ T cells due to ongoing viral replication, ultimately resulting in clinical immunodeficiency and death. While antiretroviral therapy (ART) can effectively suppress viral replication, reservoirs of virally infected cells persist for life, requiring indefinite pharmacological suppression treatment (1–3). These reservoir cells harbor chromosomally integrated genome-intact viral DNA but can effectively evade immune surveillance, likely due to limited viral gene expression and, possibly, an intrinsic ability to resist immune-mediated killing. A rare subset of individuals able to maintain undetectable levels of plasma viremia in the absence of ART has been identified as outliers of natural HIV disease progression (4, 5). Known as elite controllers (ECs), these individuals make up < 1% of the total people living with HIV-1 (PLWH), although their frequency is up to 4-fold higher in females compared with males (6–8). Antiviral immune activity in these persons has been mostly linked to HIV-1–specific T cell responses (9–11), as evidenced by a strong enrichment of ECs with protective HLA class I alleles that restrict cytotoxic T cell activity (12, 13); however, alternative components of the human immune system may also contribute to antiviral immune defense in ECs (14).

Despite undetectable levels of plasma viremia, persisting reservoirs of virally infected cells remain detectable in the vast majority of ECs, although the frequencies of such cells are typically much smaller compared with PLWH on suppressive ART (15, 16). Moreover, the integration sites of genome-intact proviruses in ECs are frequently biased toward heterochromatin regions, likely as a result of immune selection pressure that selectively targets more transcriptionally active proviruses integrated in accessible chromatin regions (17, 18). Most prior studies have analyzed persisting viral reservoirs of ECs in peripheral blood mononuclear cells (PBMC) due to the ease and limited invasiveness of sample collection. However, the vast majority of viral reservoir cells, by some estimates > 95% (19), are located in lymphoid tissues, arguably because germinal centers in lymph nodes and gastrointestinal lymphoid tissues offer an immune privileged location that can protect infected cells from host immune activity (20, 21). Whether and how viral reservoir cells from ECs can persist and longitudinally resist immune elimination in lymph nodes remains uncertain at present.

In this study, we characterized and compared the HIV-1 reservoir cells in matched PBMCs and inguinal lymph node mononuclear cells (LNMCs) from 2 ECs from whom longitudinal samples over 9–10 years of continuous, drug-free viral control were available. We observed that intact proviruses in lymph nodes from ECs were smaller in size, less phylogenetically diverse, and longitudinally declining over time, all suggesting that the lymph node viral reservoir in ECs is vulnerable to host immune responses.

## Results

### Study participants.

**Figure 1:**
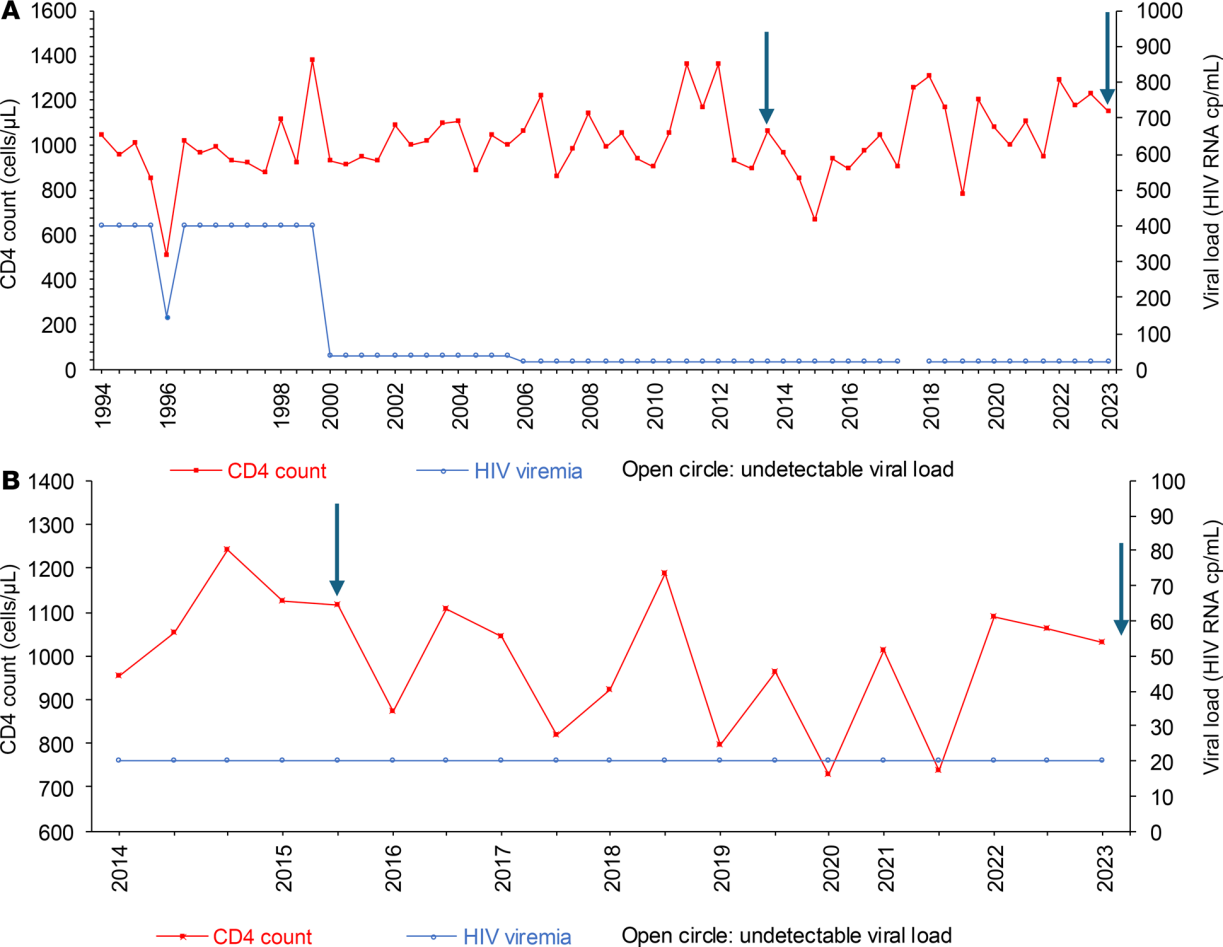
CD4^+^ T cell count and viral loads in the 2 ECs under investigation. (**A** and **B**) Graphs of viral loads and CD4^+^ T cell counts for Participant 1 (**A**) and Participant 2 (**B**). Open circles indicate RNA viral loads below the level of detection for the assay. Arrows indicate the time points at which the samples were collected.

We focused our analysis on 2 ECs who were identified at the Lausanne University Hospital and followed over time. The first participant is female and was diagnosed with HIV-1 in 1992 at the age of 31. She never received ART and has maintained undetectable viremia since diagnosis, except for a single transient viral load of 148 copies/mL in 1996 (Figure 1A). Paired PBMC and inguinal LNMC samples were collected in 2013 and 2023, 21 and 31 years after diagnosis, respectively. Participant 1 has the protective HLA B*57 allele, and isolated viral sequences were classified as being clade A1 (Supplemental Table 1; supplemental material available online with this article; https://doi.org/10.1172/jci.insight.197308DS1). The second participant is also female and was diagnosed with HIV-1 in 2011, at the age of 44. She has also never been treated and has maintained undetectable viremia since diagnosis (Figure 1B). Paired PBMC and inguinal LNMC samples were collected in 2015 (4 years after diagnosis), and additional PBMC samples were collected in 2024 (13 years after diagnosis). Participant 2 also possesses the protective HLA B*57 allele, and isolated proviral sequences were classified as clade K (Supplemental Table 1). In both study participants, HIV-1 acquisition had likely occurred via sexual transmission.

### Quantitative evolution of intact proviruses.

To characterize the proviral reservoir landscape within the 2 types of tissues, we used near full-length individual proviral sequencing (FLIP-Seq) (22) and matched integration site and proviral sequencing (MIP-Seq) (23) assays. These techniques allow for the identification of both genome-intact and defective proviruses with their corresponding chromosomal locations at single-genome resolution.

**Figure 2:**
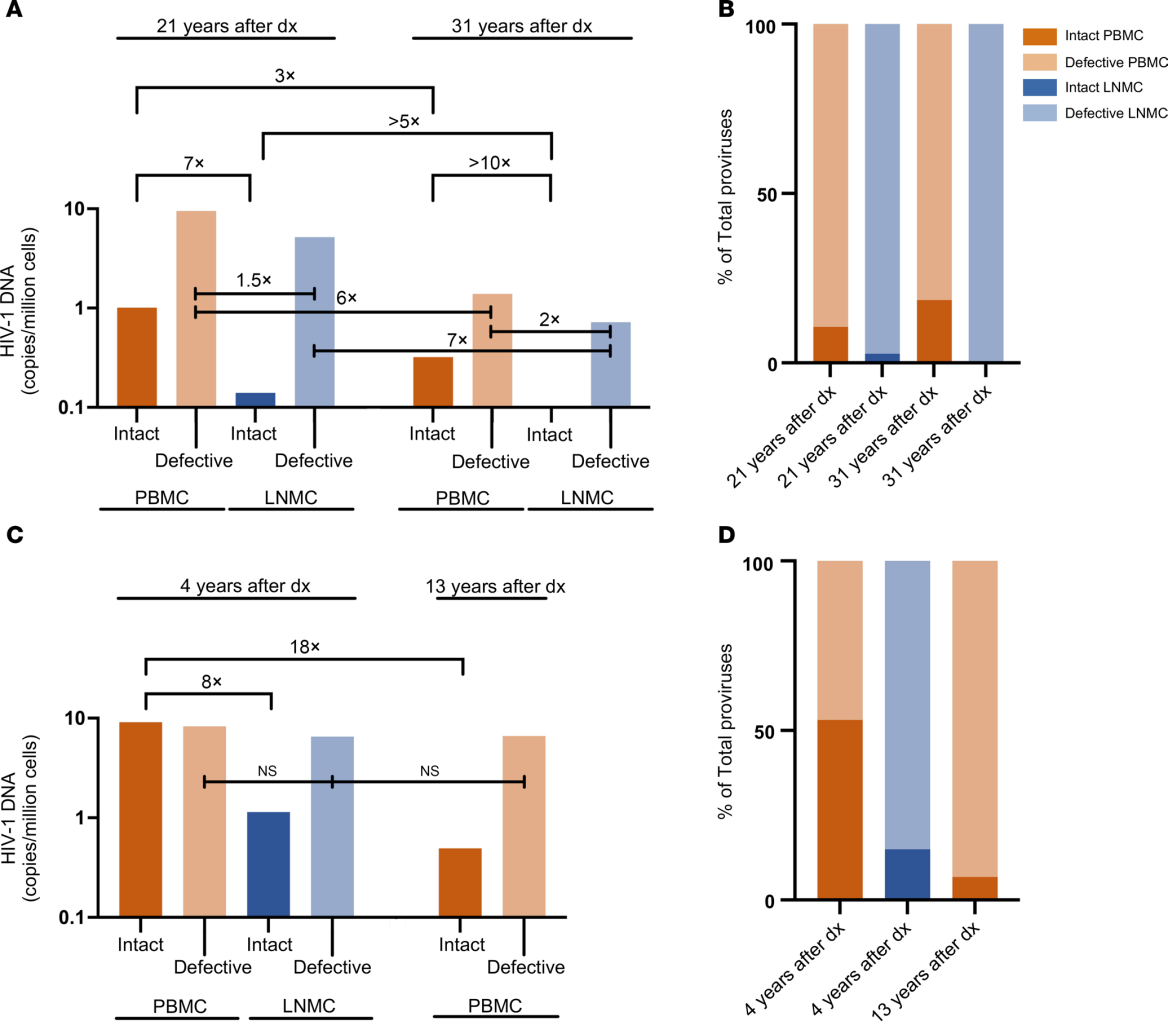
Quantification of the proviral reservoirs. (**A** and **C**) Relative frequencies of intact and defective HIV-1 proviral sequences per million cells in PBMC and LNMC samples of Participant 1 (**A**) and Participant 2 (**C**) across time points. “Dx” denotes diagnosis. Horizontal bars indicate calculated fold changes between sample types. (**B** and **D**) Percentages of intact and defective HIV-1 proviral sequences out of the total number of proviral sequences detected in PBMC and LNMC samples of Participant 1 (**B**) and Participant 2 (**D**) across time points.

In the PBMC sample collected 21 years after diagnosis from Participant 1, 13 of the total 122 proviruses detected in 13 million cells were identified as genome-intact. At the same time point in the LNMC sample, only 2 of the 75 total proviruses detected in 14 million cells were genome intact, corresponding to a 7-fold smaller intact reservoir in the LNMC sample. Thirty-one years after diagnosis, 5 intact proviruses of 27 total proviruses were detected in 16 million PBMCs, consistent with a 3-fold decline (from 1.01 copies/million PBMCs to 0.32 copies/million PBMCs) relative to the initial sample. In the lymph node sample collected 31 years after diagnosis, not a single intact provirus was detected out of 13 total proviruses in 18 million cells assayed, indicating at least a 10-fold lower frequency of intact proviruses in the lymph node compared with the contemporaneous blood sample and at least a 5-fold lower frequency of intact proviruses compared with the initial lymph node sample collected 10 years earlier (Figure 2, A and B, and Supplemental Table 2).

For Participant 2, we detected 68 intact proviruses out of 128 total proviruses in 7 million PBMCs from a sample collected 4 years after diagnosis, reflecting a relative frequency of 9.22 genome-intact proviruses per million PBMCs. In the LNMC sample from the same time point, 10 intact proviruses were detected out of 67 total proviruses in 9 million analyzed cells (1.14 genome-intact proviruses/million cells), consistent with an approximately 8-fold lower frequency of intact proviruses in LNMCs relative to PBMCs. At the subsequent analysis time point 9 years later, we detected 9 intact proviruses out of 132 proviruses in a total of 18 million PBMCs analyzed (0.49 intact copies/million), consistent with a reduction by approximately 18-fold relative to the initial analysis time point (Figure 2, C and D, and Supplemental Table 2).

### Clonality and integration site analysis.

**Figure 3:**
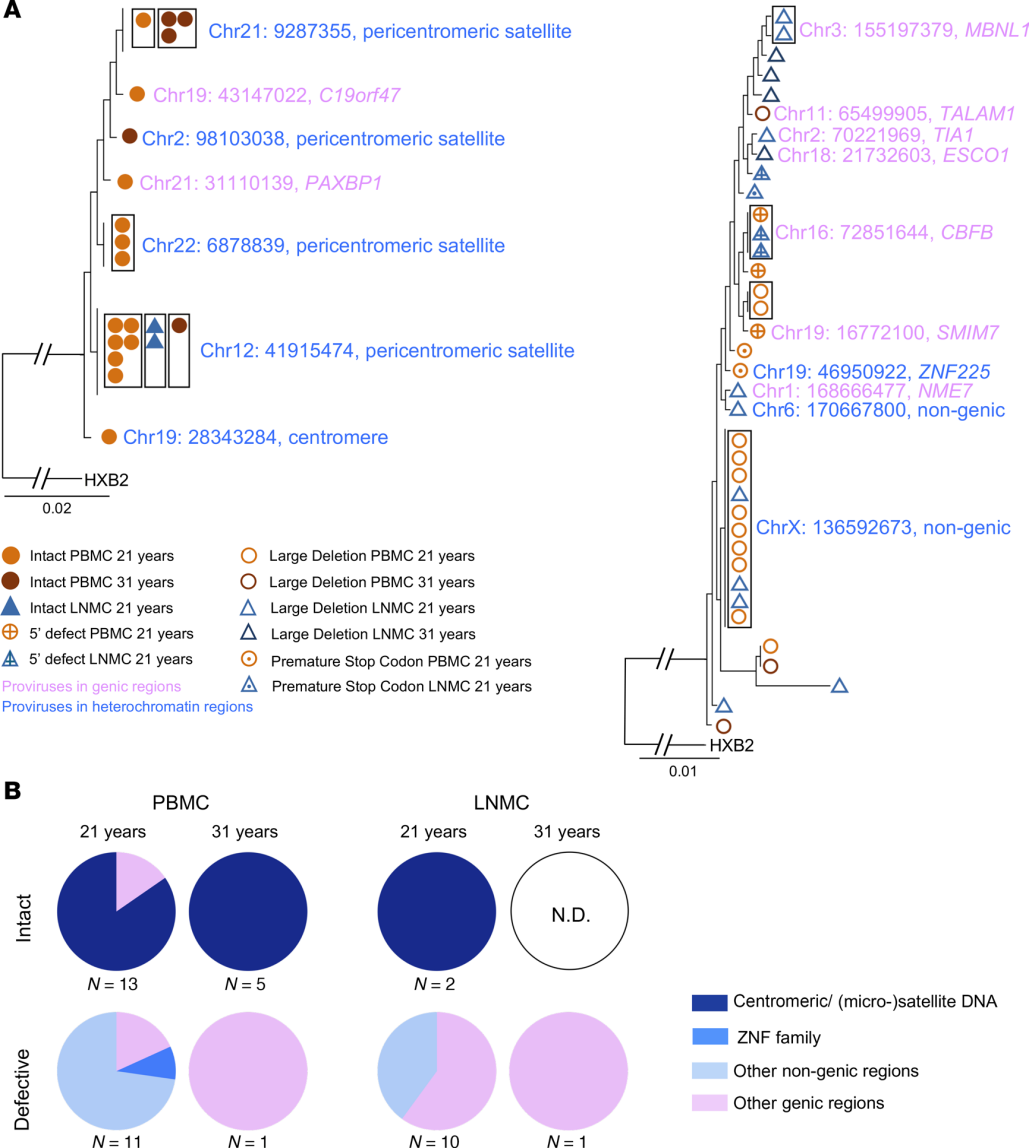
Integration site profile of proviruses detected in participant 1. (**A**) Phylogenetic trees of intact proviruses and defective proviruses with their integration sites. Clonality is indicated by a black box surrounding the sequence symbols. HXB2 is the HIV-1 reference sequence. Integration site locations are reported from the T2T reference human genome according to the UCSC human genome browser and NCBI RefSeq. Genic versus nongenic locations are indicated by text highlight color. (**B**) Pie charts showing the proportions of intact and defective HIV-1 sequences located in genic, centromeric/satellite, ZNF family, or other nongenic DNA regions in PBMC and LNMC across time points. There were no intact sequences identified in the 31-year time point, so it is labeled as not detectable (N.D.).

To better characterize the proviral reservoir cell composition, we utilized integration site loop amplification (24) to identify chromosomal locations of individual genome-intact proviruses, as described in our prior work (23). In PBMC samples collected from Participant 1 at 21 years after diagnosis, we detected 2 large clusters of sequence-identical intact proviruses, one of which was integrated into pericentromeric satellite DNA on Chromosome 12 and the other into pericentromeric satellite DNA on the upper arm of Chromosome 22. Genic chromosomal locations were only observed for 2 of the 13 individual genome-intact proviruses. Only 2 intact proviruses were detected in the lymph node at this same time point, and both of these were a part of the large clone integrated into satellite DNA on Chromosome 12 that was also detected in the PBMC sample. In the second time point collected 10 years later, the same clone in satellite DNA on Chromosome 12 was detected again in PBMCs, highlighting the ability of this clone to persist over 10 years. At the same time, we observed 3 clonal genome-intact proviruses integrated in a pericentromeric nongenic region in Chromosome 21; one member of this clone was also originally detected in the first time point. All intact proviruses detected at this time point were in nongenic locations. No intact proviruses were detected in the LNMC samples at all in this second time point (Figure 3, A and B, and Supplemental Table 3A). Defective proviruses were detected across both time points and tissue compartments, and they were primarily integrated into genic regions of the genome. Only 1 large clone of proviruses with a large deletion at the 3’ LTR end of the proviral genome was located in a nongenic region on Chromosome X, and this clone was detected in both the PBMC and LNMC samples collected at the first time point (Figure 3A).

**Figure 4:**
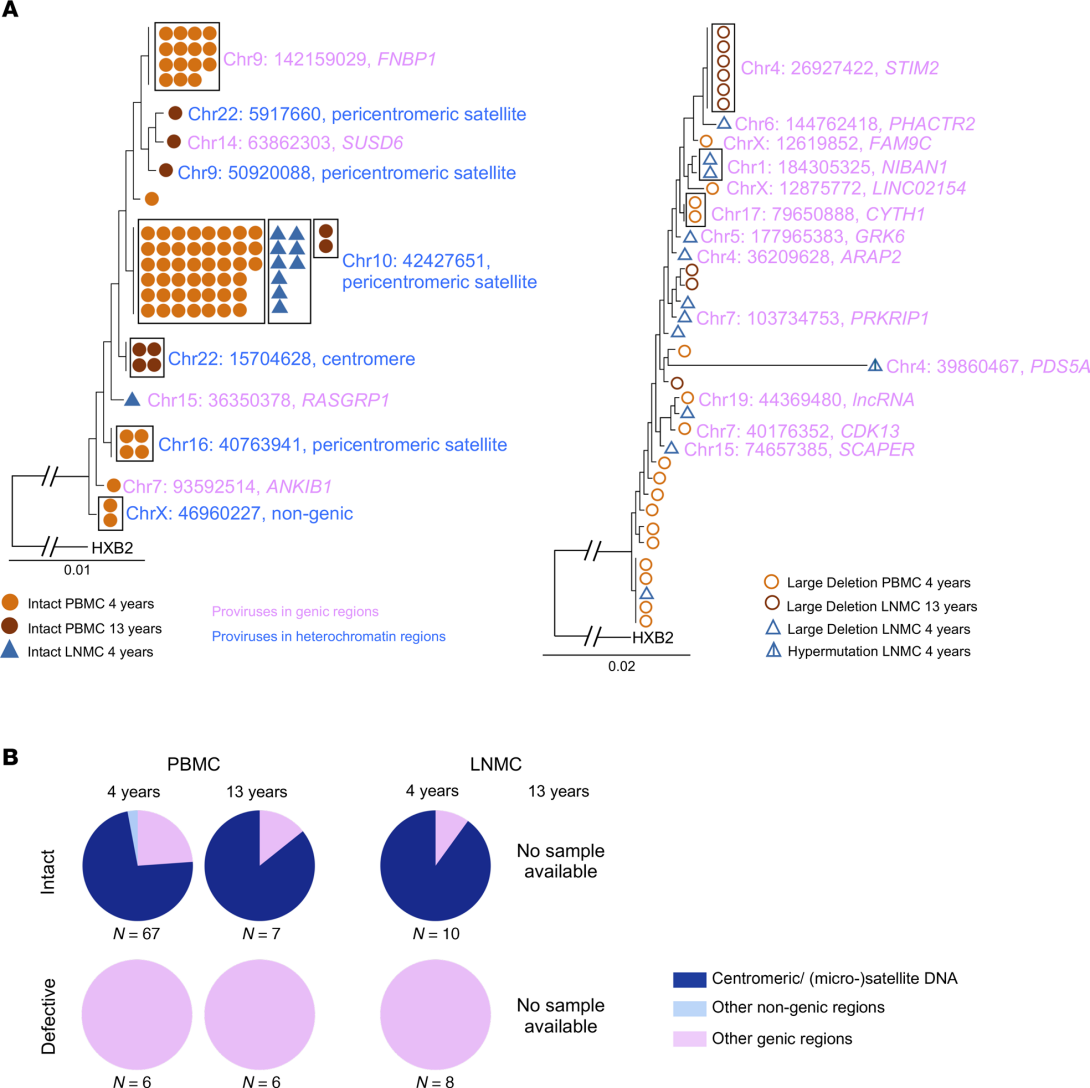
Integration site profile of proviruses detected in participant 2. (**A**) Phylogenetic trees of intact proviruses and defective proviruses with their integration sites. Clonality is indicated by a black box surrounding the sequence symbols. HXB2 is the HIV-1 reference sequence. Integration site locations are reported from the T2T reference human genome according to the UCSC human genome browser and NCBI RefSeq. Genic versus nongenic locations are indicated by text highlight color. (**B**) Pie charts showing the proportions of intact and defective HIV-1 sequences located in genic, centromeric/satellite, or other nongenic DNA regions in PBMC and LNMC across time points. It should be noted that there was no LNMC sample available for the 13-year time point.

In Participant 2’s PBMC sample collected 4 years after diagnosis, we detected 4 different clones of reservoir cells with intact proviruses. Three of them, including the largest one, were integrated in the pericentromeric noncoding regions of Chromosome 10 and Chromosome 16, and in the nongenic region of Chromosome X. The second largest clone was integrated into the *FNBP1* gene on Chromosome 9; this gene encodes for a protein involved in activating the focal adhesion kinase (FAK) and may promote cancer cell survival through activation of the PI3K/AKT/mTOR pathway (25). In the LNMC sample of the same time point, all but 1 intact provirus was detected in the pericentromeric region of Chromosome 10, the same chromosomal location (Chr10: 42427651) that made up the largest clone in the PBMC sample. At the second time point, 13 years after diagnosis, 2 more intact proviruses were detected within the clone integrated in Chromosome 10, consistent with clonal persistence over 9 years of viral control. Furthermore, at the second time point, an additional intact proviral clone was detected in the centromeric region of Chromosome 22. Interestingly, the large clone located in the *FNBP1* gene on Chromosome 9 did not persist in the second time point at our level of detection (from 15 sequences in 7 million PBMCs 4 years after diagnosis to 0 sequences in 18 million PBMCs 13 years after diagnosis) (Figure 4A). Consistent with Participant 1 and other previously characterized ECs, the majority of defective proviruses detected were integrated into genic locations along the genome. In particular, several clones of proviruses with large deletions were detected, which were integrated in the *NIBAN1* gene on Chromosome 1, the *CYTH1* gene on Chromosome 17, and the *STIM2* gene on Chromosome 4 (Figure 4, A and B, and Supplemental Table 3B). Persistence of defective proviruses in genic regions suggests that these proviruses are under limited immune selection pressure, presumably because sequence defects in HIV-1 proviruses reduce vulnerability to host immune recognition mechanisms.

### HLA and CTL escape mutations.

Since viral mutational escape in CTL epitopes has been previously reported in ECs (26, 27), we investigated the sequence diversification in HLA-B*57 epitopes of intact proviruses to infer immune selection pressure by cytotoxic T cells. In Participant 1, three of the 12 HLA-B*57 restricted epitopes displayed mutations previously linked to CTL immune escape (ISPRTLNAW, TSTLQEQIGW, KAAFDLSFF).This included the A146P mutation preceding the immunodominant ISW9 (ISPRTLNAW) gag epitope, which was detected in all genome-intact proviruses and has been linked to inhibiting CTL epitope processing, as it prevents proper peptide trimming by aminopeptidase I (28) (Supplemental Table 4A). The immunodominant TW10 (TSTLQEQIGW) gag epitope displayed a glycine (G) to arginine (R) switch at position 9; however, the canonical CTL escape mutation T3N G9A/E (29) was not detected. No sequence variation was detected in the immunodominant KF11 (KAFSPEVIPMF) gag epitope. The majority of CTL epitopes restricted by alternative HLA class I alleles displayed WT sequences. This, combined with the absence of major canonical variants in the TW10 and KF11 epitopes, suggest that infected cells from blood and lymph nodes in Participant 1 remain largely susceptible to CTL-dependent immune pressure.

Participant 2 displayed very little sequence adaptation to CTL immune pressure. Of the 12 HLA-B*57 CTL escape epitopes, only 2 epitopes (QASQEVKNW, KAAFDLSFF) were found to contain variants (S3T N8G; A2G) that could reflect escape from T cell immunity (Supplemental Table 4B). However, the immunodominant TW10, KF11, and ISW9 epitopes did not contain any mutations at all. The majority of HLA-A*2 and HLA-A*30 and HLA-B*15, including the immunodominant HLA-A*2–restricted SL9 (SLYNTVATL) gag epitope, were WT or contained mutations that are not known to be linked to CTL escape. Thus, Participant 2 displays limited evidence for CTL-driven sequence evolution in proviral sequences from blood and lymph nodes.

## Discussion

Despite sustained global efforts, the UNAIDS 2025 targets for reducing HIV incidence, prevalence, and mortality remain unmet; annual new infections are estimated at approximately 1.3 million, and nearly one-quarter of all people living with HIV remain untreated, highlighting the limitations of current treatment and prevention strategies and the urgent need for the development of curative approaches to end the HIV epidemic (30). ECs provide compelling evidence that spontaneous, immune-mediated control of HIV-1 is achievable, thus representing a model for HIV control without ART (14). Unraveling the virological and immunological mechanisms that underlie this drug-free control of viral replication may offer valuable insights for the development of curative interventions that could be applicable to the broader population of people living with HIV. In this study, we longitudinally analyzed persisting viral reservoirs in blood and lymph node samples from 2 individuals who maintained drug-free viral control for extended periods of time. Our data show that, in these 2 study participants, intact proviruses displayed more limited frequencies in lymph nodes compared with blood, progressively decreased over time, and were frequently restricted to chromosomal locations with inhibitory and repressive chromatin features. Together, these results support the hypothesis that proviruses persisting in ECs are subject to sustained and effective immune selection pressure.

Lymphoid tissues have long been regarded as major anatomical sites for persistence of HIV reservoir cells, arguably because they provide an immune-privileged environment that protects infected cells from host immune attacks (31, 32). This view is supported by foundational studies demonstrating reduced cytolytic CD8^+^ T cell activity within lymphoid tissues compared with blood (33), which has been attributed to structural and immunological features that limit immune effector cell access to lymphoid tissue compartments, specifically to germinal centers (34). However, the analysis of viral reservoirs in lymph nodes has been difficult due to the invasive nature of lymph node sampling, and the ethical and logistical challenges associated with harvesting lymphoid tissue biopsies sufficient for the analysis of HIV infected cells (35). Serial excisional lymph node sampling in the current study provided rare and critical insight into reservoir dynamics in ECs. In both ECs examined, intact proviral sequences in lymph nodes were 7- to 10-fold less abundant than in blood. In Participant 1’s second time point, not a single intact provirus was detected in over 18 million lymph node cells assayed, suggesting that the true difference might exceed a 10-fold difference. Furthermore, in both participants, there were fewer total infected cells in LNMCs than in PBMCs. These results suggest that inguinal lymphoid tissues in ECs do not represent privileged sites of viral persistence and that antiviral immune responses are effective in targeting and controlling viral reservoirs in lymphoid tissues from ECs. Previous work has indeed demonstrated that ECs can mount robust cytolytic CD8^+^ T cell responses within lymphoid tissues, supporting the conclusion that the immune system of ECs exerts sustained pressure against the reservoir cell pool residing in lymphoid tissues (36). Of note, the majority of intact proviruses detected in LNMCs from the 2 ECs were clonally expanded sequences that were also detected in PBMCs, suggesting a multicompartment dissemination of infected cell clones across blood and lymphoid tissues, as previously shown for study participants on suppressive ART (37–39).

Longitudinal studies in individuals on ART suggest a progressive decrease of reservoir cells over time; however, this decline is slow and, according to some studies, predominantly occurs during the initial 7 years; afterward, frequencies of reservoir cells often plateau, continue to decline, or in rare cases, increase due to clonal proliferation of infected cells (40–42). Longitudinal studies of viral reservoirs in ECs are difficult as the low frequency of viral copies necessitates deeper investigations with larger cell quantities. In our work, detailed, single-molecule sequencing of individual proviruses, paired with chromosomal integration site profiling, permitted a longitudinal analysis of the viral reservoir in great detail. Of note, our data show that there is a continuous, selective decline of intact proviruses across both tissue compartments over time. In Participant 1, the intact proviral reservoir declined 3-fold in PBMCs and 5-fold in LNMCs after 10 years. In Participant 2, an 18-fold reduction was seen in PBMCs over 9 years. The more modest decline observed in Participant 1 could be attributed to the extended duration of viral suppression (21 years) prior to sample collection. In this study participant, intact proviruses were almost entirely integrated in heterochromatin locations, where they arguably are in a deeper state of latency, and less vulnerable to host immune activity that could further decrease their frequency. Nevertheless, the continued decline in Participant 1’s reservoir after more than 2 decades of control strongly suggests a continuous targeting of viral reservoir cells by the immune system of ECs that is sustained for years after the initial infection and occurs at a rate that seems faster than the decay half-life of intact proviruses in persons receiving long-term suppressive ART (42, 43). Notably, the more profound longitudinal decline of intact proviruses in Participant 2 was also associated with qualitative changes in the viral reservoir profile. In particular, we observed that frequencies of intact proviruses in heterochromatin locations, already encompassing more than 77% of all intact proviruses at the earlier time point of investigation, increased to more than 88% at the time of final analysis. These observations are consistent with progressive immune selection of intact proviruses in heterochromatin locations (17, 44) and reinforce the concept that viral reservoirs in ECs are frequently limited to rare cells harboring intact proviruses in nonpermissive chromosomal regions. However, it should be noted that a small proportion of intact proviruses remained integrated within genic regions in our 2 study persons, possibly because they may actively resist immune elimination. Alternatively, these intact proviruses in genic locations may be transcriptionally repressed by other not fully characterized mechanisms, such as hypermethylation in the viral promoter region.

There are several limitations of this study that warrant consideration and highlight the need for continued research in this area. One major limitation is the use of inguinal lymph node tissue samples, as it is unclear if our observations here generalize to other lymphoid tissues, such as mesenteric lymph nodes. The other major limitation of this study is the sample size of 2 participants, although the consistency of our findings among those 2 study participants strengthens our conclusion. Our results provide an important initial step toward a better understanding of longitudinal reservoir cell dynamics in lymphoid tissues from ECs; however, expanding this analysis to additional ECs and individuals on long-term ART is essential. Taken together, this study highlights the immunological vulnerability of HIV-1 reservoirs in ECs and advances insight of specific immune imprints in the viral reservoir profile of ECs. Further understanding of how host immune responses in ECs interface and interact with persisting viral reservoirs may inform the development of functional cure strategies aiming to induce drug-free control of HIV in larger numbers of people living with HIV in the future.

## Methods

### Sex as a biological variable.

Our study focuses on the analysis of 2 previously identified ECs, both of whom are female. While sex has been identified as having an impact on elite control, for this study sex was not considered as a variable for outcome parameters.

### Study participants and cell samples.

Participants were recruited by the Lausanne University Hospital. PBMC and LNMC samples were collected at defined time points and processed and cryopreserved using standard procedures.

### DNA extraction and ddPCR.

DNA extraction from PBMCs and LNMCs was performed using commercial kits (Qiagen DNeasy Blood and Tissue Kit, #69504). Droplet Digital PCR (Bio-Rad) was performed to quantify cell numbers and total HIV-1 DNA using a previously described procedure (22).

### Proviral sequencing.

Near FLIP-Seq was performed on DNA diluted to single-genome levels and amplified using Invitrogen Platinum Taq with nested primers spanning near-full-length HIV-1 (HXB2 coordinates 638-9632), using protocols and primers previously published (22). MIP-Seq was performed on genomic DNA that underwent whole-genome amplification, as previously described (23). For this purpose, DNA in each well was subjected to whole genome amplification with phi29 polymerase (#150345; Qiagen REPLI-g Single Cell Kit), per the manufacturer’s protocol. Following this unbiased whole genome amplification, DNA from each well was split and separately subjected to near full-length viral sequencing and integration site analysis, as described below. For both FLIP-Seq and MIP-Seq, agarose gel electrophoresis was used to visualize PCR products. All near full-length amplicons were subjected to Illumina MiSeq sequencing at the MGH DNA Core facility. Resulting short reads were de novo–assembled using Ultracycler v1.0 and aligned to HXB2 to identify large deleterious deletions (<8,000 bp of the amplicon aligned to HXB2), out-of-frame indels, premature/lethal stop codons, internal inversions, or packaging signal defects (≥15 bp insertions and/or deletions relative to HXB2), using an automated in-house pipeline written in Python programming language (https://github.com/BWH-Lichterfeld-Lab/Intactness-Pipeline; commitID: 0c59371a5e37f6a25300b5394eb7d843cba7cb5a). The presence/absence of APOBEC3G/3F-associated hypermutations was determined using the Los Alamos National Laboratory (LANL) HIV Sequence Database Hypermut 3.0 program. Viral sequences that lacked all mutations listed above were classified as “genome-intact” sequences. Sequence alignments were performed in Geneious Prime (https://www.geneious.com/) using MAFFT alignment (https://mafft.cbrc.jp/alignment/software) and visualized using Highlighter plots (https://www.hiv.lanl.gov/content/sequence/HIGHLIGHT/highlighter_top.html). Viral sequences were considered clonal if they had completely identical consensus sequences; single nucleotide variations in primer binding sites were not considered for clonality analysis. Clades of intact HIV-1 proviral sequences were determined using the LANL HIV Sequence Database Recombinant Identification Program.

### Integration site analysis.

Integration Site Loop Amplification Assay (ISLA) was used to obtain integration sites, as previously described (24). DNA produced by whole genome amplification was used as the template. PCR products of the ISLA reaction were subjected to next-generation sequencing using Illumina MiSeq. MiSeq paired-end FASTQ files were demultiplexed; small reads (142 bp) were then aligned simultaneously to the T2T human reference genomes (45) and HIV-1 reference genome HXB2 using bwa-mem (46). Biocomputational identification of integration sites was performed according to previously described procedures (23). The final list of integration sites and corresponding chromosomal annotations was obtained using Ensembl (v114, www.ensembl.org), the UCSC Genome Browser (www.genome.ucsc.edu), and GENCODE (v48, www.gencodegenes.org). Repetitive genomic sequences harboring HIV-1 integration sites were identified using RepeatMasker (www.repeatmasker.org).

### HLA and CTL escape mutations.

The list of CTL epitopes and their corresponding documented escape variants was obtained using the LANL HIV Molecular Immunology Database Best-defined CTL/CD8^+^ Epitope Summary (last updated 2024-01-12, https://www.hiv.lanl.gov/content/immunology/tables/optimal_ctl_summary.html). Genome-intact proviral sequences were translated using the LANL HIV Sequence Database Gene Cutter (https://www.hiv.lanl.gov/content/sequence/GENE_CUTTER/cutter.html) and aligned to HXB2 in Geneious Prime (https://www.geneious.com/) to visualize the epitope sequences.

### Study approval.

The IRB committees of the Mass General Brigham and the Lausanne University Hospital approved this study. Study participants gave written informed consent.

### Data availability.

Due to confidentiality concerns of the study participants, full-length proviral sequencing data cannot be publicly released but will be made available to investigators upon reasonable request and after signing a data sharing agreement. Values for all data points in graphs are reported in the Supporting Data Values file.

## Author contributions

Participant recruitment and biological specimen collection were contributed by MC, AM, and MP. Analysis of viral DNA was contributed by SKM, CMN, and CGC. Integration site analysis was contributed by SKM, CMN, LC, CGC, and ICR. Bioinformatic analysis was contributed by CG. Data analysis, interpretation, and presentation were contributed by SKM, CMN, CG, ML, and XGY. Writing and preparation of manuscript and figures were contributed by SKM, CMN, AM, ML, MP, and XGY. Project supervision was contributed by ML, MP, and XGY. Co–first authorship order was determined alphabetically by last name for SKM and CMN.

## Funding support

This work is the result of NIH funding, in whole or in part, and is subject to the NIH Public Access Policy. Through acceptance of this federal funding, the NIH has been given a right to make the work publicly available in PubMed Central.

- MP is supported by Swiss National Science Foundation Grant 320030_200912 and by Freedom Forever.
- ML is supported by NIH grants AI130005, DK120387, AI152979, AI155233, AI135940, and AI176579.
- XGY is supported by NIH grants AI155171, AI116228, AI078799, HL134539, DA047034, amfAR ARCHE (grant no. 110393-72-RPRL), and the Bill and Melinda Gates Foundation (INV-002703).
- ML and XGY are members of the DARE, ERASE, PAVE, and BEAT-HIV Martin Delaney Collaboratories (UM1 AI164560, AI164562, AI164566, and AI164570).

## Supplementary Material

Supplemental data

Supporting data values

## Version 1 10/28/2025

In-Press Preview

## Version 2 12/08/2025

Electronic publication
