# Supplementary material for: Viral reservoir characteristics in lymphoid tissues of HIV-1 elite controllers
Source: JCI Insight. 2025 Oct 28;10(23):e197308. doi: 10.1172/jci.insight.197308 (PMC12890494; doi:10.1172/jci.insight.197308)
Supplement: Supplemental data [file jciinsight-10-197308-s115.pdf]

Supplemental Table 1- Demographic information. Clinical profile of each participant.

	Sex	Age	Year of Diagnosis	HIV Clade	HLA-A	HLA-B	HLA-C
Participant 1	Female	64	1992	A1	01:01/11:01	35:01/57:41	04:01/06:02
Participant 2	Female	58	2011	K	02:01/30:02	15:31/57:03	16:01/18:02

Supplemental Table 2- Total number of cells assayed. Number of million cells assayed for each participant at each timepoint for each tissue type. The last two columns show the exact numbers of genome-intact and defective proviruses detected in each sample type. “Dx” denotes diagnosis.

Participant	Timepoint	Sample Type	Total Cells Assayed (million)	# Intact Proviruses Detected	# Defective Proviruses Detected
Participant 1	21 years after dx	PBMC	13	13	109
		LNMC	14	2	73
	31 years after dx	PBMC	16	5	22
		LNMC	18	0	13
Participant 2	4 years after dx	PBMC	7	68	60
		LNMC	9	10	57
	13 years after dx	PBMC	18	9	123
		LNMC	NA	NA	NA

Supplemental Table 3- Summary of Integration Sites. For Participant 1 (**A**) and Participant 2 (**B**).

A

Sequence No.	Viral Seq. Intactness	Chrom.	Position	Strand	Gene	Intron/ Exon	Repeats
1	Intact	chr12	41915474	-	pericentromeric satellite	n/a	LINE: L1MC4
2	Intact	chr19	28343284	+	centromere	n/a	Satellite: ALR/Alpha
3	Intact	chr19	43147022	+	*C19orf47*	Intron	
4	Intact	chr2	98103038	-	pericentromeric satellite	n/a	
5	Intact	chr21	31110139	+	*PAXBP1*	Intron	
6	Intact	chr21	9287355	+	pericentromeric satellite	n/a	
7	Intact	chr22	6878839	+	pericentromeric satellite	n/a	Satellite: HSATIB
8	Premature Stop Codon	chr19	46950922	+	*ZNF225*	Intron	
9	5' defect	chr16	72851644	-	*CBFB*	Intron	LINE: L1ME3F
10	5' defect	chr19	16772100	+	*SMIM7*	Intron	SINE: AluJb
11	Large Deletion	chr1	168666477	-	*NME7*	Intron	
12	Large Deletion	chr11	65499905	-	*TALAM1*	Exon	
13	Large Deletion	chr18	21732603	+	*ESCO1*	Intron	
14	Large Deletion	chr2	70221969	-	*TIA1*	Exon	
15	Large Deletion	chr3	155197379	-	*MBNL1*	Intron	
16	Large Deletion	chr6	170667800	-	non-genic	n/a	
17	Large Deletion	chrX	136592673	-	non-genic	n/a	Retroposon: SVA_A

B

Sequence No.	Viral Seq. Intactness	Chrom.	Position	Strand	Gene	Intron/ Exon	Repeats
1	Intact	chr10	42427651	+	pericentromeric satellite	n/a	Satellite: HSATIII
2	Intact	chr14	63862303	-	*SUSD6*	Intron	
3	Intact	chr15	36350378	+	*RASGRP1*	Intron	
4	Intact	chr16	40763941	+	pericentromeric satellite	n/a	Satellite: HSATII
5	Intact	chr22	15704628	-	centromere	n/a	Satellite: ALR/Alpha
6	Intact	chr22	5917660	+	pericentromeric satellite	n/a	
7	Intact	chr7	93592514	+	*ANKIB1*	Intron	
8	Intact	chr9	142159029	-	*FNBP1*	Intron	
9	Intact	chr9	50920088	+	pericentromeric satellite	n/a	Satellite: HSATII
10	Intact	chrX	46960227	-	non-genic	n/a	LTR: MLT1B
11	Large Deletion	chr1	184305325	+	*NIBAN1*	Intron	
12	Large Deletion	chr15	74657385	+	*SCAPER*	Intron	LINE: L1M4
13	Large Deletion	chr17	79650888	+	*CYTH1*	Intron	LINE: L1ME1
14	Large Deletion	chr19	44369480	+	*lncRNA*	Intron	LTR: ERV3-16A3_I
15	Large Deletion	chr4	36209628	+	*ARAP2*	Intron	SINE: MIR
16	Large Deletion	chr4	26927422	+	*STIM2*	Intron	LINE: L2a
17	Large Deletion	chr5	177965383	-	*GRK6*	Intron	LTR: MLT1I
18	Large Deletion	chr6	144762418	-	*PHACTR2*	Intron	
19	Large Deletion	chr7	40176352	-	*CDK13*	Intron	LINE: L1PB3
20	Large Deletion	chr7	103734753	-	*PRKRIP1*	Intron	SINE: AluY
21	Large Deletion	chrX	12619852	+	*FAM9C*	Exon	
22	Large Deletion	chrX	12875772	+	*LINC02154*	Intron	LINE: L1MA9
23	Hypermut	chr4	39860467	+	*PDS5A*	Intron	

Supplemental Table 4- Summary of CTL escape mutations across different HLA alleles. For Participant 1 (**A**) and Participant 2 (**B**), the table shows the numbers of cytotoxic T lymphocyte (CTL) escape mutations, and the corresponding wild-type (WT) epitope sequences with observed variant forms. Escape mutations are indicated by amino acid substitutions, insertions, or deletions compared to WT sequences.

A

HLA	Number of CTL escape epitopes analyzed	Epitopes containing an escape mutation	WT Epitope	Variant
HLA-A*01	1	0		
HLA-A*11	11	5	ACQGVGGPGHK	--------S--
			SVITQACPK	---K-----
			AVDLSHFLK	-F--GF---
			IIATDIQTK	M----L---
			QVPLRPMTYK	---V------
HLA-B*35	12	5	VPVWKEATTTL	----RD-D---
			NSSKVSQNY	NNSQ-----
			HPVHAGPIA	--PQ----P
			HPDIVIYQY	N-E------
			IPLTEEAEL	V---A----
HLA-B*57	12	3	TSTLQEQIGW	--------R-
			AISPRTLNAW	PV--------
			KAAFDLSFF	-G---G--
HLA-C*04	1	0		

B

HLA	Number of CTL escape epitopes analyzed	Epitopes containing an escape mutation	WT Epitope	Variant
HLA-A*02	17	4	RGPGRAFVTI	F---Q--YAT
			PLTFGWCYKL	-------F--
			VLEWRFDSRL	--K-V-----
			ALVEICTEM	--T------
HLA-A*30	10	1	KQNPDIVIY	IK--E----
HLA-B*15	18	3	SFNCGGEFF	----R----
			WRFDSRLAF	-V------L
			FQTKGLGISY	-LH-G-----
HLA-B*57	12	2	QASQEVKNW	-CT----G-
			KAAFDLSFF	-G-V---H-
HLA-C*18	4	0		
